# Multiple eccrine hidrocystomas on the upper lip: a case report

**DOI:** 10.1186/1757-1626-2-9291

**Published:** 2009-12-09

**Authors:** Jose Aneiros-Fernandez, Jose A Gonzalez-Saavedra, Salvador Arias-Santiago, Mercedes Caba Molina, Francisco O'Valle, Jose Aneiros Cachaza

**Affiliations:** 1 Department of Pathology. University Hospital San Cecilio, Granada, Spain; 2 Department of Dermatology. Baza Hospital, Granada, Spain; 3 Department of Dermatology. University Hospital San Cecilio, Granada, Spain

## Abstract

**Background:**

Multiple eccrine hidrocystomas of de upper lip are bening cystic lesions that are associated with a chronic course. It is reported in the literature as a rare pathology.

**Case report:**

We describe the case of a 60-year-old woman who was referred to the dermatology department for presenting multiple lesions translucent papular asymptomic two years of evolution on the upper lip. Increase in size in summer and physical exercise, improving winter.

**Conclusion:**

To make the diagnosis of multiple eccrine hidrocystomas is necessary clinical and histopathological findings, taking different lines of treatment.

## Introduction

Eccrine hidrocystomas are cystic tumor the sweat glands ducts, are relatively rare and account less than 1% of sumitted cutaneous biopsies [1]. They are more frequent in fermales between 30 and 70 years of age and can occur as solitary or multiple cyst. Multiple eccrine hidrocystoma occur more frecuently around the eyes, cheeks, nose and less frecuently on the upper lip [2].

## Case Report

**Figure 1:**
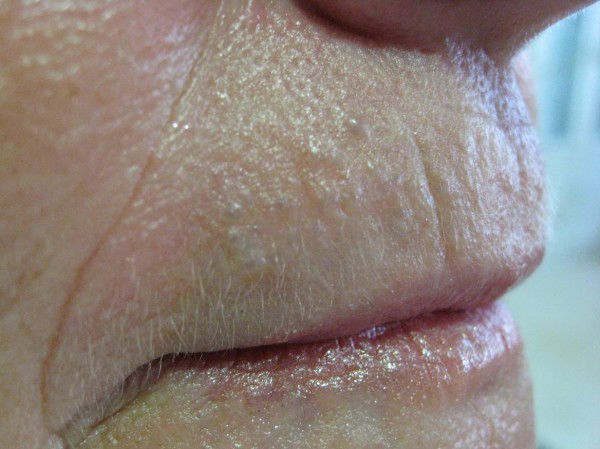
**Multiple to numerous cystic papules lesions, ranging from 2 to 3 mm in diameter upper lip**.

**Figure 2:**
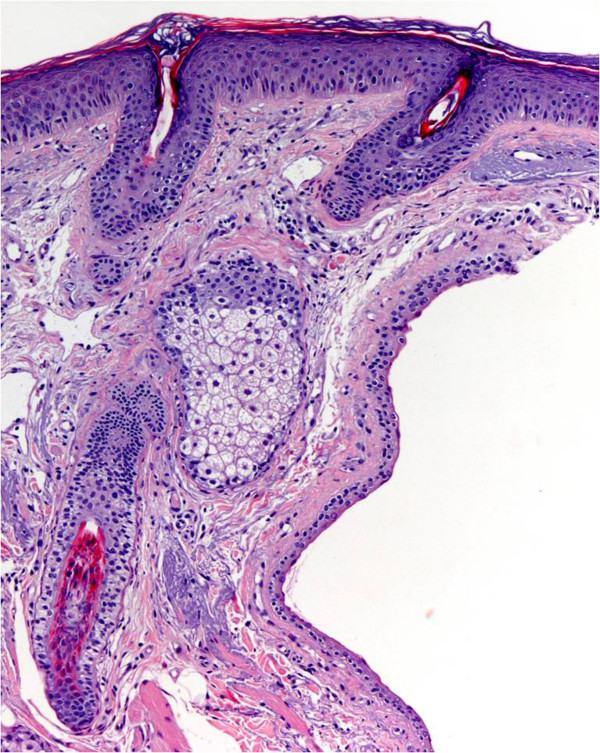
**Low magnification (×10) revealed one cyst in the thickness of the dermis composed of 2 layers of cuboidal cells (Hematoxiline eosine)**.

**Figure 3:**
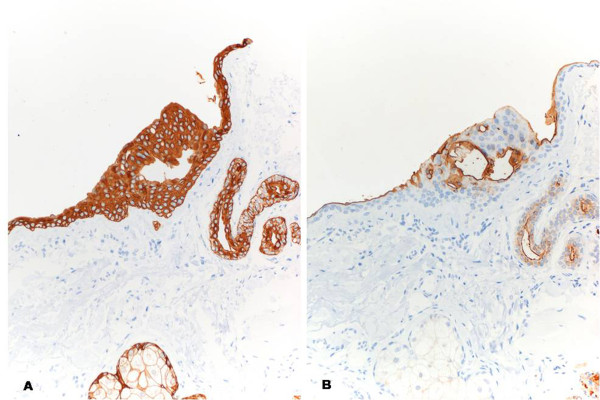
**Inmunohistochemistry presented positivity high molecular weight cytokeratins (A)(×40) and CEA (B) (×40)**.

A 60-year-old woman caucasic who was referred to the dermatology department for presenting multiple lesions translucent papular asymptomic two years of evolution on the upper lip. Increase in size in summer and physical exercise, improving winter (Fig 1). Excision was performed in one of the lesions. The histopathologic showed one cyst in the thickness of the dermis. The cyst wall composed of 2 layers of cuboidal cells with eosinophilic cytoplasm that secrete into the cyst, without decapitation (Fig 2). No evidence of PAS-positive. Inmunohistochemistry presented positivity for high molecular weight cytokeratins and CEA, and negative for GCDFP-15 (Fig 3). A diagnosis of multiple eccrine hydrocystoma was made.

## Discussion

The eccrine hidrocystoma has been considered to be true ectatic eccrine ducts of the dermal portion. The occurrence of these injuries was caused by retention of sweat favored by increased temperature and physical exercise [3]. There is an increased incidence in hyperthyroid patients, possibly related to hyperhidrosis. When the presentation is simply a cyst it may be mistaken with epidermal inclusión cyst, mucoid cyst and hidrocystoma apocrine. From the clinical point of view el eccrine hidrocystoma and apocrine hidrocystoma are difficult to distinguish; to be diagnosed histopathology is necessary. Apocrine hidrocystoma usually presented as multiple cysts and may have abundant papillary proyections into the cyst cavity or only have a simple apocrine type lining cell with large cytoplasm, PAS-positive diastase resistant granules, showing the beheading events of decapitation surrounded by myoepithelial cells [4]. However the eccrine hidrocystomas are usually unilocular showing two layers of epitelial cuboidal without decapitation events or presence of myoepithelial cells. Have been described associated with Graves Basedow disease, Goltz-Gorlin and Schopf-Schulz-Passarge syndromes.

In regard of treatment, when are unique lesions performed surgical excision when are multiple lesions several treatments have been described with favorable response; tópica 1% atropine or scopolamine creams, with a 585 mm flashlamp-pupel pulse dye laser and botulim toxim [5,6].

Eccrine hidrocystoma is not a serius injury but it is a mainly problem cosmetic.

## Consent

Written informed consent was obtained from the patient for publication of this case report and accompanying images. A copy of the written consent is available for review by the Editor-in-Chief of this journal.

## Competing interests

The authors declare that they have no competing interests.

## Authors' contributions

JAF wrote the initial draft of and helped revise the manuscript. MCM, JAS, SAS and FO obtained consent from the patients and helped revise the manuscript. JA assisted with manuscript revision. All authors read approved the final manuscript.
